# Oral pharmacological management of Bertolotti syndrome presenting as chronic low back pain – A case report and review of literature

**DOI:** 10.1016/j.jor.2024.10.028

**Published:** 2024-10-21

**Authors:** Saarim Bari, Varun Menon, Shankar Bhuvanesh

**Affiliations:** a School of Medicine, University of Central Lancashire, Preston, PR1 7BH, United Kingdom; b Cumberland Infirmary, North Cumbria Integrated Care, Carlisle CA2 7HY, United Kingdom

**Keywords:** Low back pain, Case report, Lumbosacral transitional vertebra, Congenital anomalies, Bertolotti syndrome

## Abstract

**Background:**

Bertolotti syndrome (BS) is often a missed cause of chronic lower back pain in young individuals, commonly associated with the presence of anomalous lumbosacral transitional vertebrae.

**Case presentation:**

A 19-year-old female with no significant medical or family history presented with persistent lower back pain localized to the gluteal region and posterolateral aspect of the left lower back. The pain worsened over time and limited their movements, including walking. A Ferguson radiograph revealed fusion of the left transverse process of the L5 vertebral segment with the left sacral ala. History, examination findings, and radiological workup confirmed the diagnosis of BS. The patient preferred conservative management, receiving oral pharmacological therapy for six weeks, along with education on preventive measures and routine exercises for postural stability. At a six-month follow-up, the patient remained asymptomatic and managed well.

**Conclusions:**

Conservative oral pharmacological treatment presents a unique and viable alternative to traditional methods for managing BS, which often involve surgery or steroids/anesthetics at the pseudo-articulation site. Given that BS is common yet underdiagnosed in young patients with chronic back pain, this report also underscores the importance of including it in differential diagnoses for chronic lower back pain in this demographic.

## 1 Introduction

First studied by Mario Bertolotti in 1917, Bertolotti syndrome (BS) is a congenital disorder mainly diagnosed in young individuals presenting with chronic and/or back pain associated with the presence of lumbosacral transitional vertebra (LSTV).^1^ It remains one of the main reasons for undiagnosed causes of low back pain in young subjects. LSTV is a congenital anomaly recognized by an abnormal enlargement of the transverse process and its subsequent articulation/fusion with the sacral ala. It mainly affects the fifth lumbar segment (L5), which fuses with the ala of the first sacral vertebra (S1) to varying degrees. It is the lumbarization of the S1 vertebra or sacralization of the L5 segment theorized to be the developmental basis of LSTV.^2^ This anomalous articulation results in the nonuniform distribution of the load exerted by the upper half of the body. Alongside, the enlarged and fused transverse processes negatively affect the biomechanics of the other joints in the vertebral column by restricting movements at the lumbosacral junction (commonly L5-S1) and simultaneously increasing movements above the affected segment.^1^^,^^2^ In the case of BS, the cause of the pain experienced by the patients is believed to be multifactorial, one of them being altered biomechanics at the affected joint. This results in straining and degeneration of the vertebral facets and intervertebral discs which may lead to compression of the emerging nerve root (commonly L5) at the corresponding intervertebral disc level, presenting with symptoms indicative of neurological deficits such as hyporeflexia and hypoesthesia along the affected dermatomyotome accompanied by low back pain with a different etiology. Castellvi and colleagues classified LSTV in 1984 based on the development of transverse processes as outlined in Table 1.^3^ We present a unique case of a young female with unilateral BS who was managed exclusively with conservative oral pharmacological therapy, which has not been documented in existing literature.

**Table 1:** Castellvi's Classification of lumbosacral transitional vertebrae^3^.

Type	Feature	Anatomical characteristics	Example
Type I	Dysplastic transverse process	(a) Unilateral or (b) bilateral presence of >19 mm wide transverse process	
Type II	Partial sacralization or lumbarization	(a) Unilateral or (b) bilateral pseudoarthrosis between the enlarged transverse process and sacral ala	
Type III	Total sacralization or lumbarization	Complete fusion of transverse process with sacral ala either (a) unilaterally or (b) bilaterally	
Type IV	Mixed	Type IIA pseudoarthrosis is present on one side and Type IIIA on the contralateral side	

## 2 Case presentation

**Fig. 1:**
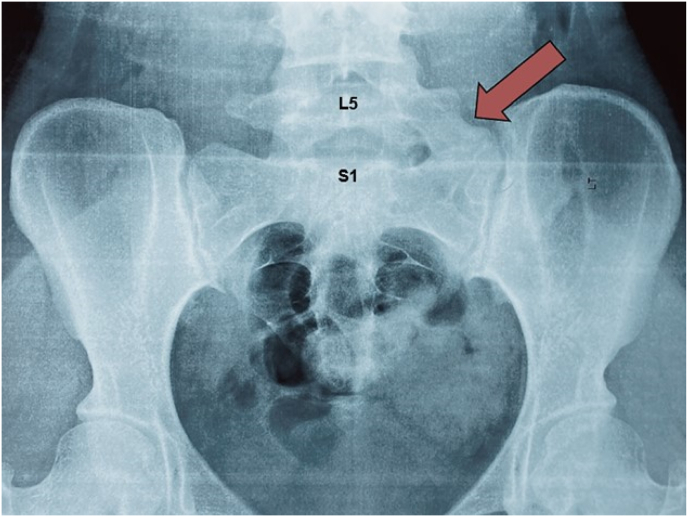
Modified Anteroposterior pelvis radiograph of the patient with central X-rays pointing towards the anterior end of the body focusing along the midline 5 cm directly below the anterior superior iliac spine, also known as Ferguson radiograph. A red arrow demonstrates abnormal overgrowth of the transverse process emerging from the L5 segment and its complete fusion with the left sacral ala consistent with type IIIA LSTV.

A young female aged 19 years presented with a complaint of persistent pain confined to the gluteal region and the posterolateral aspect of their left lower back. It was an aching and dull pain that rated 6/10 on the adult pain rating scale. It began five months previously and had worsened with time. The patient was primarily concerned about the pain she experienced when she attempted to walk and extend the hip, resulting in mobility issues. Initially, the pain was managed by self-administration of over-the-counter analgesics before the patient presented to the clinic. Past medical history and family history were not significant for any condition. There was no history of smoking, alcohol consumption, or illicit drug abuse. On assessment, the pain site lacked erythema, swelling, tenderness, and other signs suggestive of inflammation. Upon physical examination of the lumbar region, a limited range of movements including axial rotation, lateral flexion, and extension were present. Lower limb reflexes, optimal muscular tone, and pulsation were preserved bilaterally. Femoral and sciatic nerve stretch tests were negative. The inflammatory markers profile showed mildly elevated C-reactive protein (CRP) levels. An anteroposterior modified Ferguson view radiograph was obtained (Fig. 1) depicting left-sided complete sacralization of the L5 vertebra consistent with Castellvi type IIIA LSTV. The radiographical findings, along with the symptoms they presented with, confirmed the diagnosis of BS. To avoid additional costs and limit radiation exposure, no advanced imaging such as a CT scan (computed tomography) or magnetic resonance imaging (MRI) scan was used at any stage of the investigation.

At first, the patient was offered fluoroscopy-guided epidural steroid injections at the pseudo-articulation site, however, they declined to be treated either this way or surgically due to personal reasons. Hence as an alternative, an oral pharmacological course of management was agreed upon that involved the administration of 4 mg (mg) of methylprednisolone once a day (OD) and a quadruple tablet formulation of trypsin (48 mg), bromelain (90 mg), rutoside trihydrate (100 mg), and diclofenac (50 mg) taken OD. Pantoprazole 80 mg OD sustained release formulation was commenced for gastroprotection. On week 4, the methylprednisolone dose was tapered to 2 mg OD to reduce the risk of glucocorticoid withdrawal syndrome. The treatment continued for six weeks, and the patient was reviewed fortnightly for treatment adherence and response. They also began to experience relief from symptoms during this treatment period. Another aspect of care that facilitated the recovery process included relevant education and precautionary measures to prevent the worsening of pain and routine exercises to aid postural stability. The patient was followed up six months later and was found to be asymptomatic with an almost full range of movements present at their lower back.

## 3 Discussion and conclusion

BS is identified by the presence of LSTV characterized by abnormal enlargement of the transverse process typically arising from the L5 lumbar segment that either fuses completely or partially with the sacral ala. The prevalence and incidence of LSTV in the general population have been reported to range between 4 % and 35.9 %, with 13 % of individuals remaining asymptomatic. This perhaps explains the low incidence rates of BS diagnosis, which range between 4 % and 8 %.^1^^,^^4^ Different diagnostic standards and imaging modalities may also lead to variations in the incidence and prevalence rates. BS is typically diagnosed in individuals aged between 20 and 40, with around 18.5 % of cases occurring in individuals under 30 years of age. For symptomatic individuals, the mean age at diagnosis is as high as 40.2 years, which is possibly a consequence of misdiagnosed low back pain until a correct diagnosis is made^5^^,^^6^

As noted in Table 2, symptoms of BS mainly include chronic low back pain typically radiating along the L5 nerve distribution if accompanied by nerve impingement and neurological deficits such as hypoesthesia. Regarding low back pain, Type II and Type IV LSTV in patients are found to be significantly associated (p < 0.05) with gluteal pain and low back pain compared to counterparts with no LSTV^7^ Limited range of mobility, flexibility, and pain exacerbation with movement may be noted in some patients, as noted in our case. Besides chronic pain, LSTV is strongly tied to degenerative changes, including vertebral facet and intervertebral disc degeneration. The prevalence of these anatomical alterations varies by the level of the joint affected and the LSTV type. Across all Castellvi types, the mean percentage prevalence of facet degeneration is noted to be 23.13 % and 32.5 % for L3/L4 and L4/L5 levels, respectively, and for disc degeneration, it is 15.16 % and 39.92 % for L3/L4 and L4/L5 levels.^8^ These degenerative changes may lead to mechanical back pain and cause nerve root irritation, giving rise to radicular manifestations such as tingling, hyporeflexia, and hypoesthesia along the dermatomyotome corresponding to the nerve being affected.^9^

**Table 2:** Summary of previous case reports.

Serial no (reference)	Year	Patient sex and age (at diagnosis)	Level, Castellvi type	Imaging used	Symptoms	Complete resolution of symptoms after
**1****^14^**	2019	Female, 14	L5-S1, Type IIA	Plain radiograph, CT scan, MRI scan CT scan, MRI scan	Chronic low back along midline radiating to the right leg/hip	Pseudoarthrectomy
	2019	Female,16	L5-S1, Type IIA		Low back pain radiating into the left hip	Pseudoarthrectomy
**2****^15^**	2018	Female, 37	L5-S1, Type IIA	CT scan	Axial chronic low back pain radiating to the left gluteal region and hip	Minimally invasive pseudoarthrectomy
**3****^16^**	2018	Female, 37	L5-S1, Type IV (left- Type III and right -Type I)	CT scan	Mechanical low back pain radiating bilaterally down the legs. Difficulty in sitting and weight bearing on the right side. Pain worse on standing and rotation	Posterior lumbar interbody fusion
**4****^4^**	2017	Female, 62	L5-S1, Type IIIA	Plain radiograph, CT scan	Chronic low back pain, hypoesthesia, and radicular pain along the L5 distribution	Conservative management with oral NSAIDs and methylprednisolone
**5****^17^**	2020	Male, 20	L5-S1, Type IIA	Plain radiograph	Non-radiating dull pain confined to the lower back. Limited rotation present at the hip	Localized steroid injection at pseudo articulation site combined with oral analgesics and physiotherapy

Plain radiographs remain the mainstay imaging modality for diagnosing BS. Modified AP Ferguson view Radiograph, is proven to be sensitive and effective at detecting the abnormal overgrowth of the transverse processes, as seen in Fig. 1. Advanced imaging such as a CT or MRI scan may be warranted if radiographs are inconclusive, the patient has a high body mass index (BMI), and when there is suspicion of nerve root compression. Since plain radiographs are sensitive in detecting the presence of LSTV in patients with BS, we recommend using advanced imaging techniques, such as CT and MRI scans judiciously. CT scans expose patients to high levels of radiation, while both CT and MRI scans can be expensive and unavailable, especially in resource-limited settings.

Several approaches to managing BS patients exist, among which conservative means remain widely acceptable. It involves routine manipulation of the affected joint and physiotherapy regimens that aim to improve the patient's posture and restore and preserve the biomechanics of the affected joint, thereby alleviating the symptoms. Alongside, pharmacological therapy, including nonsteroidal antiinflammatory drugs (NSAIDs) and other analgesics, can be trialed initially, which has been proven to be more effective at relieving chronic pain when combined with exercise and physiotherapy.^10^^,^^11^ Next in the treatment arsenal is imaging-guided or non-imaging-guided local administration of corticosteroids and/or anesthetic agents via injection at the pseudo-articulation or articulation site. This offers precise administration of the drug at the pain site and is known to confer symptom-free benefits in the long term. These injections can be given as a single-dose or multiple-dose regimen depending on the treatment response.

Burham (2010) reported a case of BS in which radiofrequency ablation (RFA) was used with routine coadministration of anesthetic and corticosteroids at the articulation site after other conservative measures had been tried and failed. This combined intervention effectively relieved the symptoms the patient had been enduring for over a decade. This suggests that RFA may be viable for cases where other non-invasive treatment options have been exhausted^12^

Surgical intervention is the last step in the management, which involves procedures such as pseudoarthrectomy, L4-S1 or L5-S1 segmental fusion, root decompression.^4^^,^^13^, ^14^, ^15^, ^16^, ^17^ Among the surgical approaches currently followed, segmental fusion across LSTV is significantly effective at short-term and long-term pain management (p = 0.037) in contrast with pseudoartherectomy or resection surgery.^18^

Santavirta and colleagues reported that although the surgical approach provides better pain relief, it remains equally effective at improving patients’ Oswestry disability index as conservative treatment. Hence, surgical treatment with either LSTV resection or segmental fusion should be judiciously offered to selected patients, depending on the location of the anomaly and presence or absence of intervertebral disc degeneration.^19^ Patients with LSTV can undergo surgery for decompression of stenosed foramen below the affected joint, while those exhibiting radicular symptoms are well-suited candidates for nerve root decompression procedures done with either an anterior or posterior approach that is more commonly followed.^20^

As noted, existing literature suggests administering corticosteroids/or/and anesthetic agents through injection at a pseudoarthrosis site or resorting to surgical intervention as the main method of management.^4^^,^^14^, ^15^, ^16^, ^17^ However, here, we demonstrate a case of BS that we successfully treated using a non-invasive and safe pharmacological approach. Therefore, we recommend trialing out other noninvasive treatment options such as oral pharmacological regimens, especially in young subjects, before resorting to any invasive intervention, including injections at the pseudoarticulation site that are uncomfortable to the patient. Also, while diagnosing young individuals presenting with chronic low back pain, BS should be considered as a differential diagnosis. Being underdiagnosed and presenting with a spectrum of clinical manifestations, it is challenging to diagnose BS, hence, suspected patients must be thoroughly investigated and examined to prevent misdiagnosis and treatment delay.

## CRediT authorship contribution statement

**Saarim Bari:** Conceptualization, Formal analysis, Resources, Investigation, Supervision, Writing – review & editing. **Varun Menon:** Formal analysis, Investigation, Visualization, Writing – original draft. **Shankar Bhuvanesh:** Visualization, Writing – original draft, All authors had full access to the data in the study and take responsibility for the integrity of the data.

## Authorship declaration

All authors listed meet the authorship criteria according to the latest guidelines of the International Committee of Medical Journal Editors. All authors declare that they have read and approved the final version of the manuscript.

## Disclosure statement

The authors declare that they have not received any financial support for this research and have no relationships that may pose a conflict of interest.

## Declarations of interest

None.

## Consent to publication

A written informed consent was obtained for publication. The consent form is available for further review by the editor of the *Journal of Orthopaedics*.

## Ethical statement

Ethical approval was not required from the author's institution as the patient information has been anonymized.

## Funding statement

This research has received no external financial support or non-financial support

## Declaration of competing interest

The authors declare that they have no known competing financial interests or personal relationships that could have appeared to influence the work reported in this paper.

## References

[1] Jancuska J.M., Spivak J.M., Bendo J.A. (2015). A review of symptomatic lumbosacral transitional vertebrae: bertolotti's syndrome. Internet J Spine Surg.

[2] Alonzo F., Cobar A., Cahueque M., Prieto J.A. (2018). Bertolotti's syndrome: an underdiagnosed cause for lower back pain. J Surg Case Rep.

[3] Castellvi A.E., Goldstein L.A., Chan D.P.K. (1984). Lumbosacral transitional vertebrae and their relationship with lumbar extradural defects. Spine.

[4] Kapetanakis S., Chaniotakis C., Paraskevopoulos C., Pavlidis P. (2017). An unusual case report of bertolotti's syndrome: extraforaminal stenosis and L5 unilateral root compression (Castellvi type III an LSTV). J Orthop Case Rep.

[5] Neelakantan S, Anandarajan R, Shyam K, Philip B. Multimodality imaging in Bertolotti's syndrome: an important cause of low back pain in young adults. BMJ Case Rep. Published online November 14, 2016:bcr2016217121 doi:10.1136/bcr-2016-217121. 10.1136/bcr-2016-217121 PMC5129031 27873760

[6] Quinlan J.F., Duke D., Eustace S. (2006). Bertolotti's syndrome.A cause of back pain in young people. J Bone Joint Surg Br.

[7] Tang M., feng Yang X., Yang S wen (2014). Lumbosacral transitional vertebra in a population-based study of 5860 individuals: prevalence and relationship to low back pain. Eur J Radiol.

[8] Hanhivaara J., Määttä J.H., Niinimäki J., Nevalainen M.T. (2020). Lumbosacral transitional vertebrae are associated with lumbar degeneration: retrospective evaluation of 3855 consecutive abdominal CT scans. Eur Radiol.

[9] Shinonara K., Kaneko M., Ugawa R., Arataki S., Takeuchi K. (2021). The effectiveness of preoperative assessment using a patient-specific three-dimensional pseudoarticulation model for minimally invasive posterior resection in a patient with Bertolotti's syndrome: a case report. J Med Case Rep.

[10] Muir J.M. (2012). Chiropractic management of a patient with low back pain and Castellvi type II lumbosacral transitional vertebrae. J Chiropr Med.

[11] Almeida DB de, Mattei T.A., Sória M.G. (2009). Transitional lumbosacral vertebrae and low back pain: diagnostic pitfalls and management of Bertolotti's syndrome. Arq Neuropsiquiatr.

[12] Burnham R. (2010). Radiofrequency sensory ablation as a treatment for symptomatic unilateral lumbosacral junction pseudarticulation (Bertolotti's Syndrome): a Case Report. Pain Med.

[13] Chang C.J., Chiu Y.P., Ji H.R., Chu C.H., Chiu C.D. (2022). Surgical interventions for Bertolotti's syndrome: case report and review of unsatisfactory cases in the literature. BMC Surg.

[14] Louie C.E., Hong J., Bauer D.F. (2019). Surgical management of Bertolotti's syndrome in two adolescents and literature review. Surg Neurol Int.

[15] Yousif S., Wood M. (2018). Minimally invasive resection of lumbosacral pseudojoint resulting in complete resolution of a lower back pain – A case report and review of Bertolotti syndrome. J Clin Neurosci.

[16] Adams R., Herrera-Nicol S., Jenkins A.L. (2018). Surgical Treatment of a Rare Presentation of Bertolotti's Syndrome from Castellvi Type IV Lumbosacral Transitional Vertebra: Case Report and Review of the Literature. J Neurol Surg Rep.

[17] Kumar J., Ali S., Zadran N., Singh M., Ahmed Z. (2020). A Rare Case of Bertolotti's Syndrome in a Young Patient: A Case Report and Literature Review. Cureus.

[18] Mikula A.L., Lakomkin N., Ransom R.C. (2022). Operative Treatment of Bertolotti Syndrome: Resection Versus Fusion. World Neurosurg.

[19] Santavirta S., Tallroth K., Ylinen P., Suoranta H. (1993). Surgical treatment of Bertolotti's syndrome. Arch Orthop Trauma Surg.

[20] Mikula A.L., Lakomkin N., Ransom R.C. (2022). Operative Treatment of Bertolotti Syndrome: Resection Versus Fusion. World Neurosurg.

